# Comparison of white-to-white measurements using four devices and their determination of ICL sizing

**DOI:** 10.1186/s40662-022-00308-z

**Published:** 2022-10-02

**Authors:** Robert Edward T. Ang, Edward Kenneth F. Reyes, Fernando Amado J. Ayuyao, Maria Isabel N. Umali, Emerson M. Cruz

**Affiliations:** 1 Asian Eye Institute, Phinma Plaza, Rockwell Center, 8Th Floor, 1200 Makati, Philippines; 2 Cardinal Santos Medical Center, 10 Wilson St., Greenhills, 1502 San Juan, Philippines

**Keywords:** ICL vault, Orbscan II, IOLMaster 700, Pentacam AXL, Caliper

## Abstract

**Background:**

To compare the measurements obtained from the Orbscan II, IOLMaster 700, Pentacam AXL, and Castroviejo caliper and their effects on calculating the recommended implantable collamer lens (ICL) size and postoperative vault measurements.

**Methods:**

This is a retrospective cross-sectional study of patients who underwent ICL surgery by a single surgeon from March 1, 2018 to July 31, 2021. Records were reviewed for the anterior chamber depth (ACD) and white-to-white (WTW) measurements obtained from the Orbscan II, IOLMaster 700, Pentacam AXL, and Castroviejo caliper (WTW only). These were used to calculate the recommended ICL size. The actual ICL size implanted, and vault measurements obtained one month postoperatively were also collected.

**Results:**

One hundred seven eyes with a mean age of 27.9 ± 7.7 years were included in the study. Mean WTW measurements were significantly different between devices (*P* < 0.0001), with the IOLMaster 700 having the highest value (12.14 ± 0.04 mm) and the caliper having the lowest value (11.45 ± 0.04 mm). Mean ACD measurements were the lowest in Orbscan II (3.12 ± 0.25 mm) and the highest in Pentacam AXL (3.16 ± 0.24 mm). The Pentacam AXL produced an ICL size similar to the Orbscan in 69.2% of eyes. The IOLMaster yielded an ICL measurement one size larger than Orbscan-based calculations in 64.5% of eyes. Using the Orbscan WTW and ACD, the desired vault of 0.25 to 0.75 mm and 0.25 to 1.00 mm was achieved in 70% and 91% of eyes, respectively. Substituting caliper WTW to IOLMaster 700 or Pentacam AXL WTW increases the percentage of achieving the desired vault to 80%, similar to the Orbscan.

**Conclusions:**

The Orbscan II, IOLMaster 700, and Pentacam AXL cannot be used interchangeably for calculating ICL sizing. Combining the WTW from caliper measurement with the ACD of the IOLMaster 700 or Pentacam AXL could improve ICL sizing and achieve a higher percentage of eyes with the desired vault.

## Background

Refractive errors can be surgically corrected either by reshaping the cornea through laser vision correction or implanting a phakic intraocular lens such as the Visian implantable collamer lens (ICL, Staar Surgical, Monrovia, CA, USA). The Visian ICL has been approved by the United States Food and Drug Administration in 2003 to be a safe and effective refractive procedure in correcting moderate to high myopia and myopic astigmatism [1, 2]. ICLs were typically only used for patients who have been disqualified from laser vision correction due to thin cornea, forme fruste or keratoconus, and refractive errors beyond the safe and acceptable range of laser vision correction because of the high cost. However, because of the high accuracy rate of refractive correction, good patient feedback, reversible procedure and its little effect on future IOL calculations [2], surgeons are expanding the indications for ICL use to patients with normal corneas or lower refractive errors and even offering the ICL option to patients qualified for laser vision correction.

Apart from accuracy in calculating the refractive correction, selection of the appropriate size of the ICL is crucial in achieving a desirable outcome since most postoperative complications associated with this surgery are related to suboptimal lens sizing. Considering that the ICL has an anterior vault design and is placed directly behind the iris which may keep the ICL in contact with the iris, it is possible that the implantation of an ICL will affect pupil dynamics under scotopic conditions or photopic conditions. In addition, implanting an oversized lens can cause a pupillary block, iris chafing with pigment dispersion, angle closure, increased intraocular pressure (IOP), and malignant glaucoma [3]. An undersized lens, on the other hand, can cause cataracts, particularly anterior subcapsular cataract, or zonular damage with dislocation of the ICL [4], and astigmatic shift due to rotation of a toric ICL [2]. The vault, which is the safe distance between the posterior surface of the ICL and the anterior capsule of the crystalline lens, is the measurable gauge of how the ICL best fits in the sulcus space. Previous studies have considered 0.25 to 0.75 mm [5–8] or 0.25 to 1.0 mm [9] as the ideal vault measurement range. Precise definitions for this ideal range have remained elusive because only a percentage of eyes with suboptimal vaults experienced adverse events [10]. Thus, it is argued that suboptimal lens sizing causing either an excessive or insufficient vault should only be considered a risk factor instead of a complication because not all eyes with vaults beyond the predefined safe range experience vault-related adverse events. [11]

Accuracy and consistency in ICL sizing continue to be topics of discussion and investigation. Various published studies showing comparisons of new devices, measurements and best practices increase the precision of vault prediction in comparison with universally accepted methods. When ordering an ICL, the manifest refraction determines the lens power. The anterior chamber depth (ACD) which is measured from the endothelium and the white-to-white (WTW) measurements determine the lens size. These parameters are entered into the STAAR Surgical Online Calculation and Ordering System (OCOS™, Staar Surgical, USA). The ACD can only be measured using imaging devices, whereas the WTW can be measured with automated devices and manually with calipers.

The Orbscan II (Bausch and Lomb, Germany) is a slit-scanning topography device that can measure the WTW and ACD. It has been in use since the 1990s for refractive surgery screening and ICL sizing measurements, but production has been discontinued since the mid-2000s. Currently, only a few units remain functional. In our practice, we have had a good experience using the Orbscan II for ICL sizing. However, we routinely perform manual caliper measurements of the horizontal limbus to obtain WTW measurements in all our patients to confirm those obtained from the Orbscan WTW because there are instances when patients have pannus, pterygium, limbal scars, or wide greyish limbal edges which have affected WTW measurements with the Orbscan II.

More recent devices used for biometry and tomography, such as the IOLMaster 700 (Carl Zeiss Meditec, Jena, Germany) and Pentacam AXL (Oculus Optikgeräte GmbH, software 1.25r15), also provide measurements for WTW and ACD. Most clinics may not have an Orbscan II but may have either a Pentacam AXL or IOLMaster 700. As surgeons may want to implant an ICL in these clinics, it is therefore important to evaluate if the raw data obtained from these devices are interchangeable and the ICL sizing calculation reproducible.

Here, we aim to compare the WTW measurements obtained manually using the caliper with the three automated machines, the Orbscan II, IOLMaster 700, and Pentacam AXL. We also evaluated whether the measurements produced by each device achieved the target ICL sizing calculations and if they are in agreement with each other. This study will help ophthalmologists choose which combination of parameters (WTW and ACD) from which device would yield the greatest percentage of eyes in the optimum vault range undergoing ICL refractive surgery.

## Methods

### Patients and methods

This study is a single-center, single-surgeon, retrospective, cross-sectional review of medical records of healthy patients who underwent refractive surgery using ICL at an ambulatory surgicenter. It was approved by the ethics review board of the institution and adhered to the tenets of the Declaration of Helsinki.

#### Data collection

A database search was performed of consecutive patients with moderate to high myopia and myopic astigmatism who underwent ICL surgery from March 1, 2018 to July 31, 2021. Patients aged 18–50 years old who underwent preoperative refractive screening for ICL surgery, and had complete preoperative caliper, Orbscan II, IOLMaster 700, and Pentacam AXL measurements were included. Data completeness was confirmed by obtaining a printout of the measurements or accessing the files saved on the respective machines. Patients who did not have a one-month postoperative follow-up with an anterior segment OCT measurement were excluded from this study. The following data were gathered from the medical records: sex, laterality of the eye, preoperative and postoperative uncorrected visual acuity at distance, manifest refraction. The WTW measured from 0° to 180° by a single examiner using the caliper was also noted. Biometric parameters including WTW, internal ACD, keratometry, and central corneal thickness were measured using the following machines: Orbscan II, IOLMaster 700, and Pentacam AXL.

The Orbscan (Bausch and Lomb, Germany) topographer was introduced in 1995. It was based on the innovative principle of measuring the dimensions of a slit-scanning beam projected on the cornea. Later, with the advent of computerized topography (1999), the Orbscan II (Orbtek, Inc.) evolved. One of its clinical applications is to use digital image processing for WTW measurements. Information about corneal shape, corneal thickness, and ACD is acquired by scanning the anterior eye segment with a slit beam. The computer automatically detects the corneal limbus (the border between the white sclera and darker iris image) by comparing gray-scale steps and calculates the corneal diameter. [12]

The IOLMaster 700 (Carl Zeiss Meditec, Jena, Germany) is the latest generation biometer in the IOLMaster series and one of the most popular biometers used in the world today. It uses swept-source optical coherence tomography capable of generating b-scans using lasers with variable wavelengths (high-frequency 1,055 nm tunable laser source) to produce biometric data such as axial length (AL), lens thickness, central corneal thickness, keratometry, pupil size, ACD, and WTW. [13]

The Pentacam AXL (Oculus Optikgeräte GmbH, software 1.25r15), introduced in 2015, consists of a Scheimpflug camera with partial coherence interferometry optical biometer. It can measure anterior segment tomography, ACD, WTW corneal diameter measurements, central corneal thickness, anterior and posterior corneal surface and aberrations, AL, and corneal topography. [13]

Ramon Castroviejo invented a graduated compass-like caliper which measured from 0 to 20 mm in 1 mm increments [14, 15]. The Castroviejo caliper is still often used in ophthalmology today and has applications in other medical branches [15]. However, its use in comparative studies of ocular dimensions has not been very encouraging, possibly because of its low resolution [16]. WTW measurements in ICL sizing calculations require sub-1 mm readings. Since our caliper's graduation markings are in 1.0 mm increments, readings were estimated to the nearest 0.1 mm reading.

Intraoperative and postoperative data obtained include the actual ICL size implanted during surgery, and the one-month postoperative vault measured using an anterior segment OCT (Visante OCT, Carl Zeiss, Germany). The vault measurements obtained were categorized into groups with a specified vault size range (< 0.1 mm, 0.1–0.24 mm, 0.25–0.50 mm, 0.51–0.75 mm, 0.76–1.00 mm, 1.00–1.25 mm and > 1.25 mm). The number and corresponding percentages of eyes falling into each group and those within the recommended vault range of 0.25–0.75 mm and 0.25–1.00 mm were also determined.

#### ICL size calculation

The Visian ICL is a plate-haptic design lens made of proprietary collamer material with a central convex/concave optical zone. This design features a forward vault intended to minimize ICL contact with the anterior capsule of the natural lens. The more recent models, VICMO (EVO), VTICMO (EVO Toric), VICM5 (EVO +), or VTICM5 (EVO + Toric), have a central port or hole measuring 0.36 mm (KS-Aquaport). The central hole was meant to obviate the need for a Nd:YAG iridotomy previously required with the older V4 model to allow physiologic aqueous humor circulation. Long-term studies investigated the safety profile of the ICL, and reports have shown generally low rates of adverse events. [17]

The Visian ICL has four different manufactured ICL sizes to accommodate normal variations of intraocular anatomy, namely 12.1, 12.6, 13.2, and 13.7 mm. [11] The EVO + and EVO + Toric models have a larger optic size (6.1 mm) than the EVO and EVO Toric, but all models have the same general design and overall diameter across the manufacturer's size variations. The ICL sizing calculation is the same for these ICL models. The online ICL size calculator determines the ICL size based on the Orbscan WTW and ACD data entered into the calculator. The surgeon receives the recommendation and makes the final decision in terms of lens size to be implanted.

ICL sizing was determined using the STAAR Surgical Online Calculation and Ordering System (OCOS™, Staar Surgical, USA). For this study, we performed six OCOS calculations using the WTW and ACD obtained from different devices: Orbscan WTW with Orbscan ACD, caliper WTW with Orbscan ACD, IOLMaster WTW with IOLMaster ACD, caliper WTW with IOLMaster ACD, Pentacam WTW with Pentacam ACD, and caliper WTW with Pentacam ACD. The ICL size obtained using the WTW and ACD measurements from the Orbscan was arbitrarily designated as the benchmark to which all other calculations were compared because this was our standard point of reference and was the basis for final selection of ICL sizing in our practice. Results obtained were validated by our supervising technician who checked for artifacts and invalid measurements. The ICL sizing determined from these different combinations of raw data were compared to the ICL size implanted per eye.

#### Surgical technique

On the day of surgery, patients were administered with Sanmyd-P (Tropicamide + Phenylephrine Hydrochloride, Santen Pharmaceutical Company, Shiga, Japan) as dilating agent and Alcaine (Proparacaine Hydrochloride, Alcon, Fort Worth, Texas, USA) as anesthetic agent to the operative eye. A Visian ICL [VICMO (EVO), VTICMO (EVO Toric), VICM5 (EVO +), or VTICM5 (EVO + Toric) model] was inserted through a small, 3.2 mm, clear corneal incision. The lens was injected through a clear corneal incision at 220° if it involved the right eye and 30° if it involved the left eye using a Staar MicroSTAAR injector (STAAR Surgical Co., Monrovia, CA, USA) and allowed to unfold slowly. The distal and proximal footplates were tucked under the iris with a modified intraocular spatula. Correct positioning of the ICL in the center of the pupillary zone was verified. Any remaining viscoelastic was meticulously irrigated out of the anterior chamber with balanced salt solution.

#### Statistical analysis

Demographic data were analyzed using SPSS Statistics for Windows, version 21 (IBM Corp., Armonk, NY, USA). Mean and standard deviation were used for continuous variables, while frequency and percentage were used for categorical variables. To determine if there were significant differences between mean diameters obtained with devices, paired t-test and Bland-Altman analyses were performed. The limits of agreement (LOA) were defined as mean ± 1.96 standard deviations of the differences between two measuring devices. *P* values of less than 0.05 were considered significant.

## Results

This study reviewed the WTW, ACD, ICL size calculations, and postoperative vault of 107 eyes of 56 patients. The mean age of the patients was 27.9 ± 7.7 years and comprised 39 females (69.6%) and 17 males (30.4%).

The caliper had the smallest mean WTW (11.45 ± 0.04 mm), while the IOLMaster had the largest mean WTW (12.14 ± 0.04 mm). Pairwise comparison showed that the mean difference was the smallest between the Orbscan (− 0.1103 ± 0.1918 mm, *P* < 0.0001) and the caliper and the largest between the Orbscan and the IOLMaster (0.5757 ± 0.1742 mm, *P* < 0.0001, Table 1).

**Table 1:** Mean corneal diameter (WTW) measurements of the Orbscan, Caliper, IOLMaster, and Pentacam

	Orbscan	Caliper	IOLMaster	Pentacam
Mean WTW (mm)	11.56 ± 0.03	11.45 ± 0.04	12.14 ± 0.04	11.77 ± 0.4

Pairwise comparison		Paired difference		*P* value
		Mean	SD	
Orbscan	Caliper	− 0.1103	0.1918	0.0001
Orbscan	IOLMaster	0.5757	0.1742	0.0001
Orbscan	Pentacam	0.2084	0.1749	0.0001

WTW = white-to-white

The Orbscan had the shallowest mean ACD (3.12 ± 0.25 mm), while the Pentacam had the deepest mean ACD measurement (3.16 ± 0.24 mm). Pairwise comparison showed that the mean difference in ACD was not significant between the Orbscan and the IOLMaster (*P* = 0.1611), while the mean difference in Orbscan and the Pentacam ACD measurements (*P* = 0.0029) was statistically significant (Table 2).

**Table 2:** Mean anterior chamber depth (ACD) measurements of the Orbscan, IOLMaster, and Pentacam

	Orbscan		IOLMaster	Pentacam
Mean ACD (mm)	3.12 ± 0.25		3.14 ± 0.24	3.16 ± 0.24

Pairwise comparison		Paired difference		*P* value
		Mean	SD	
Orbscan	IOLMaster	0.0125	0.0917	0.1611
Orbscan	Pentacam	0.0337	0.1144	0.0029

**Fig. 1:**
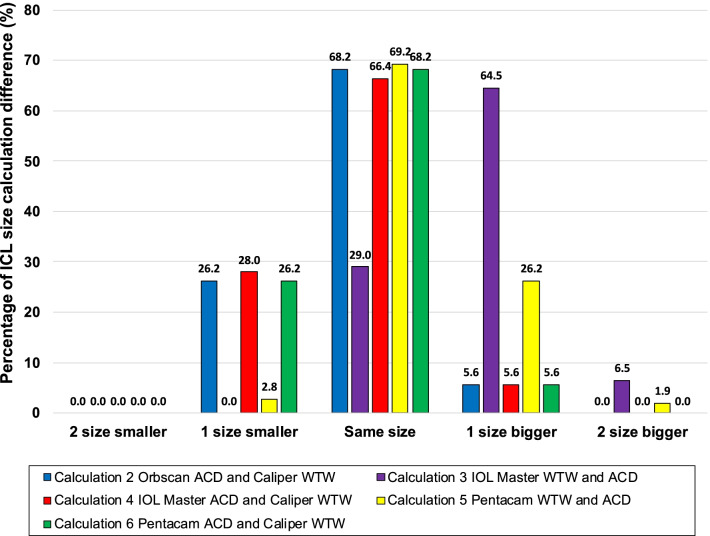
Percentage of ICL size calculation difference with reference to Orbscan WTW and ACD ICL size calculation. ACD, anterior chamber depth; ICL, implantable collamer lens; WTW, white-to-white

OCOS calculations obtained using Orbscan ACD and caliper WTW, IOLMaster ACD and caliper WTW, Pentacam WTW and ACD, and Pentacam ACD and caliper WTW yielded the highest percentage of eyes giving the same ICL size as the OCOS calculation using the Orbscan WTW and ACD (68.2%, 66.4%, 69.2%, and 68.2%, respectively). In contrast, the highest percentage of OCOS calculation suggesting a different size from the Orbscan WTW and ACD was from the IOLMaster ACD and WTW, with 64.5% of eyes yielding an ICL measurement one size larger than the reference size obtained using Orbscan WTW and ACD. For calculations where the ICL size was not the same as those obtained with Orbscan WTW and ACD, using the caliper for WTW tended to recommend smaller lenses, whereas using the Pentacam and IOLMaster WTW tended to recommend an ICL with a larger size (Fig. 1).

**Fig. 2:**
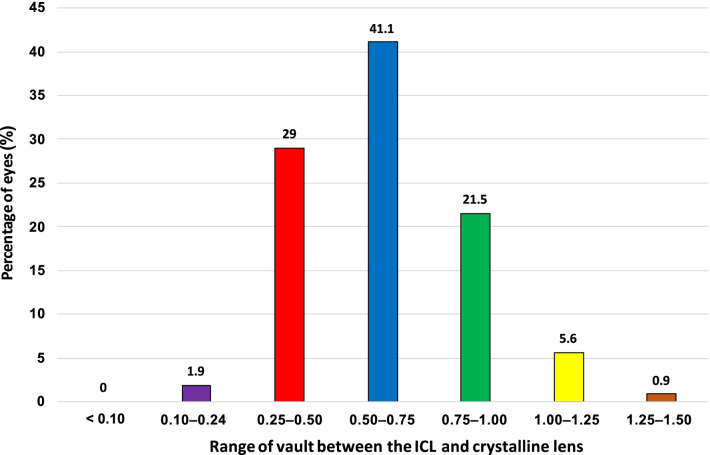
Postoperative vault at one-month follow-up

**Fig. 3:**
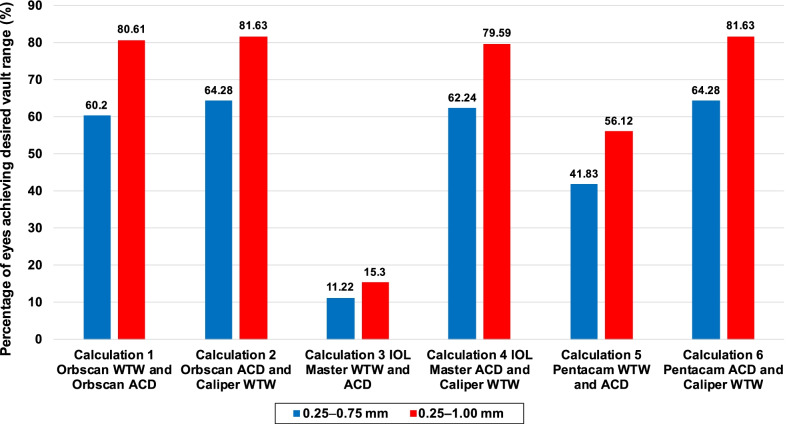
Percentage of eyes achieving the desired vault wherein the ICL implanted matched the OCOS calculations from different devices. ICL, implantable collamer lens; OCOS, online calculation and ordering system

Seventy percent (n = 75) of eyes were within 0.25 to 0.75 mm vault, while 91% (n = 98) of eyes were within 0.25 to 1.00 mm vault. The shallowest vaults were 0.16 mm and 0.24 mm, while the deepest vault was 1.27 mm (Fig. 2). OCOS calculations obtained from Orbscan WTW and ACD, Orbscan ACD and caliper WTW, IOLMaster ACD and caliper WTW, and Pentacam ACD and caliper WTW produced the highest percentage of eyes which were within the acceptable vault range of 0.25 to 0.75 mm or 0.25 to 1.0 mm. In contrast, using ACD and WTW purely from IOLMaster produced the lowest percentage of ICL size calculations that would achieve the desired vault (Fig. 3). Comparing the subvault and paired devices, results showed that the combination of IOLMaster WTW and ACD tended to recommend an oversized ICL across all vault ranges while the Pentacam WTW and ACD recommended an oversized ICL in the 0.26 to 1.00 mm vault range  (Table 3).

**Table 3:** Subvault analysis and paired devices

Vault size (mm) (N = 107)		Ordered ICL	Orbscan WTW and ACD		Orbscan ACD and Caliper WTW		IOLMaster WTW and ACD		IOLMaster ACD and Caliper WTW		Pentacam WTW and ACD		Pentacam ACD and Caliper WTW	
		Mean ± SD	Mean ± SD	*P* value*	Mean ± SD	*P* value*	Mean ± SD	*P* value*	Mean ± SD	*P* value*	Mean ± SD	*P* value*	Mean ± SD	*P* value*
< 0.25	4	12.35 ± 0.29	12.75 ± 0.30	0.10	12.35 ± 0.29	1.00	12.90 ± 0.35	0.00	12.35 ± 0.29	1.00	12.75 ± 0.30	0.06	12.35 ± 0.29	1.00
0.26 to 0.50	29	12.56 ± 0.30	12.66 ± 0.36	0.26	12.60 ± 0.34	0.63	13.15 ± 0.26	0.00	12.60 ± 0.34	0.63	12.84 ± 0.38	0.00	12.60 ± 0.34	0.63
0.51 to 0.75	44	12.69 ± 0.40	12.75 ± 0.40	0.11	12.64 ± 0.42	0.15	13.17 ± 0.36	0.00	12.62 ± 0.44	0.05	12.90 ± 0.44	0.00	12.64 ± 0.44	0.16
0.76 to 1.00	23	12.89 ± 0.34	12.93 ± 0.41	0.16	12.77 ± 0.42	0.02	13.37 ± 0.24	0.00	12.77 ± 0.42	0.02	13.10 ± 0.39	0.00	12.75 ± 0.42	0.02
> 1.00	7	13.03 ± 0.29	13.03 ± 0.29	1.00	13.03 ± 0.29	1.00	13.49 ± 0.27	0.00	13.03 ± 0.29	1.00	13.10 ± 0.39	0.36	13.03 ± 0.29	1.00

*ACD* = anterior chamber depth; *ICL* = implantable collamer lens; *WTW* = white-to-white. *t-test

**Fig. 4:**
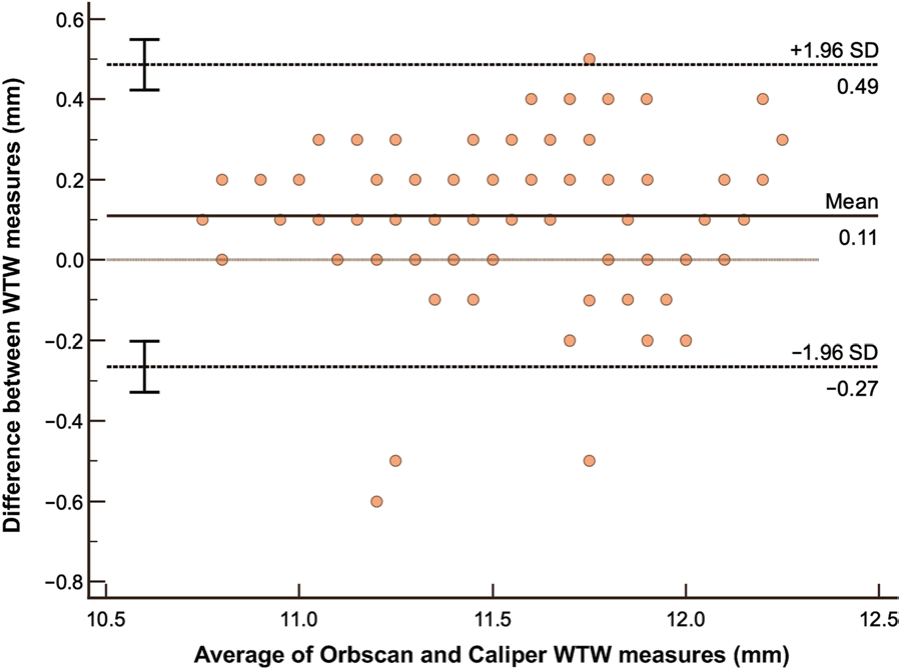
Bland-Altman analysis comparing WTW measurement using Orbscan versus Caliper. WTW, white-to-white

**Fig. 5:**
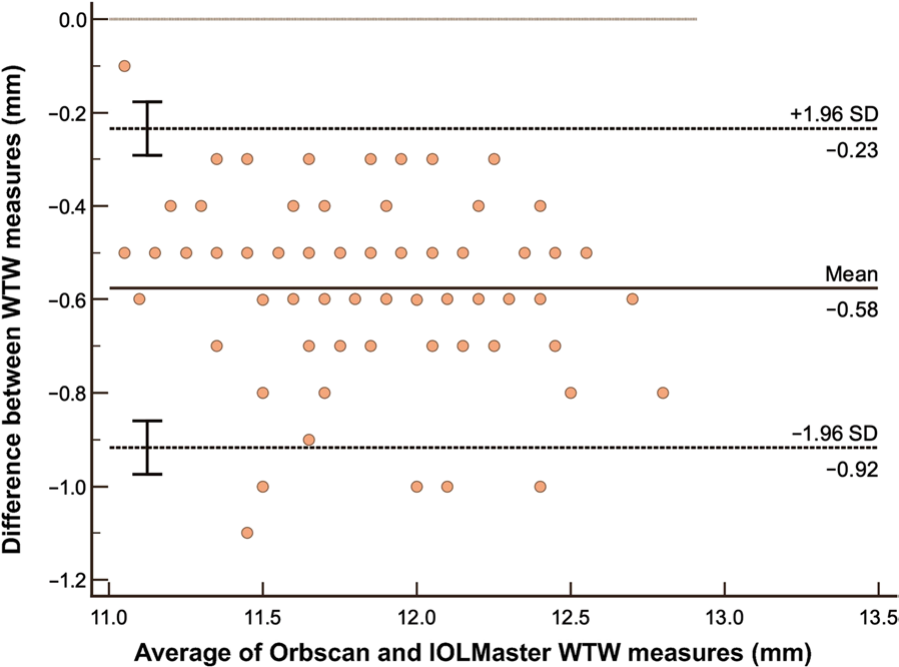
Bland-Altman analysis comparing WTW measurement using Orbscan versus IOLMaster. WTW, white-to-white

**Fig. 6:**
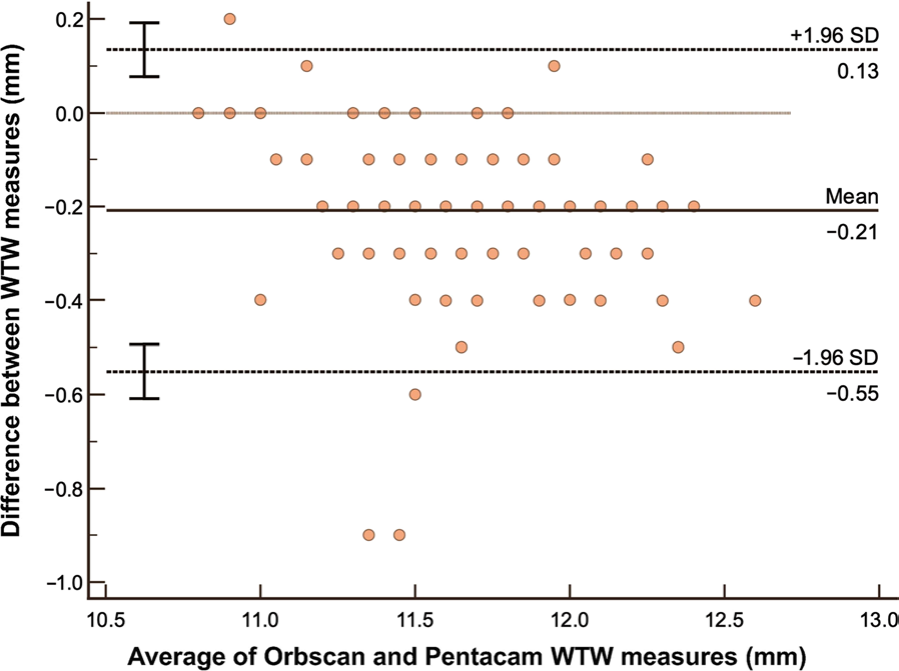
Bland-Altman analysis comparing WTW measurement using Orbscan versus Pentacam. WTW, white-to-white

The summary of LOA between devices are shown in Table 4. Bland-Altman analysis between Orbscan and caliper (Fig. 4) on WTW measure showed a mean difference 0.11 mm and LOA of − 0.27 to 0.49 mm and a high correlation coefficient of 0.8557 implying good agreement. The figure also shows that almost all data points are within the LOA. Orbscan versus IOLMaster shows a high correlation of 0.8890, but a higher systematic bias of − 0.58. Although the LOA is slightly tighter, it can be observed that the line of equality is even outside the LOA (Fig. 5). Orbscan against Pentacam shows a systematic bias of − 0.21, but also has a high correlation of 0.8985. The line of equality is within the LOA, while its limit is from − 0.55 to 0.13 mm (Fig. 6). Among the three pairwise comparisons, Orbscan to caliper has the lowest systematic bias of just 0.11, has most of the data points within LOA, and many of the data points near the line of equality.

**Table 4:** Summary of limits of agreement (LOA) between devices

	Bias (95% CI)	LOA low limit (95% CI)	LOA upper limit (95% CI)	Pearson r (95% CI)
WTW analysis				
Orbscan vs. Caliper	0.11 (0.07 to 0.15)	− 0.27 (− 0.33 to − 0.20)	0.49 (0.42 to 0.55)	0.8557 (0.80 to 0.90)
Orbscan vs. IOLMaster	− 0.58 (− 0.61 to − 0.54)	− 0.92 (− 0.97 to − 0.86)	− 0.23 (− 0.29 to − 0.018)	0.8890 (0.84 to 0.92)
Orbscan vs. Pentacam	− 0.21 (− 0.24 to − 0.17)	− 0.55 (− 0.61 to − 0.49)	0.13 (0.8 to 0.19)	0.8985 (0.85 to 0.93)
ACD analysis				
Orbscan vs. IOLMaster	− 0.01 (− 0.03 to 0.01)	− 0.19 (− 0.22 to − 0.16)	0.17 (0.14 to 0.20)	0.9288 (0.90 to 0.95)
Orbscan vs. Pentacam	− 0.03 (− 0.06 to − 0.01)	− 0.26 (− 0.30 to − 0.22)	0.19 (0.15 to 0.23)	0.8906 (0.84 to 0.92)

*CI* = confidence interval; *WTW* = white-to-white; *ACD* = anterior chamber depth

**Fig. 7:**
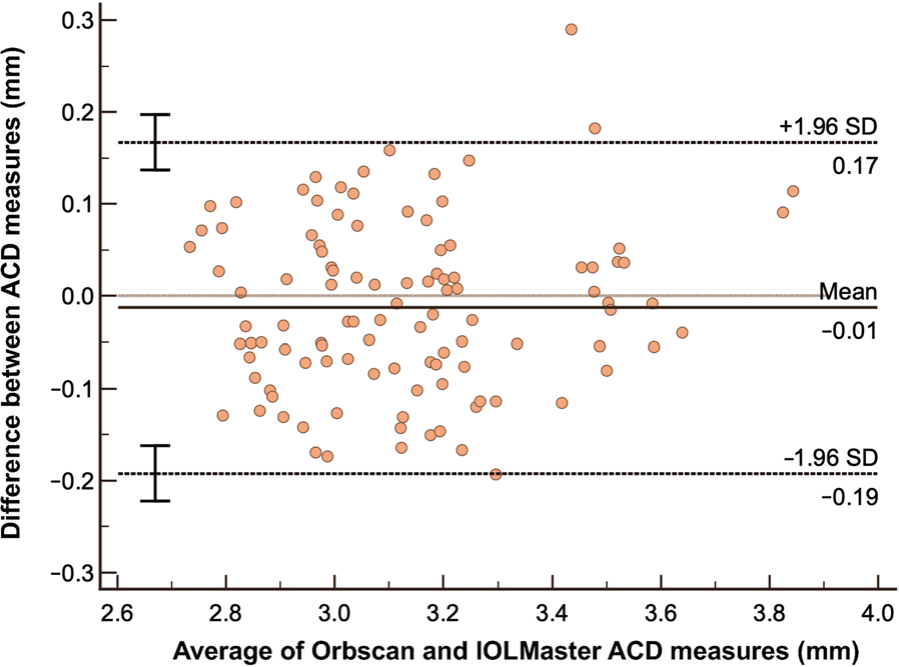
Bland-Altman analysis comparing ACD measurement using Orbscan versus IOLMaster ACD Analysis. ACD, anterior chamber depth

**Fig. 8:**
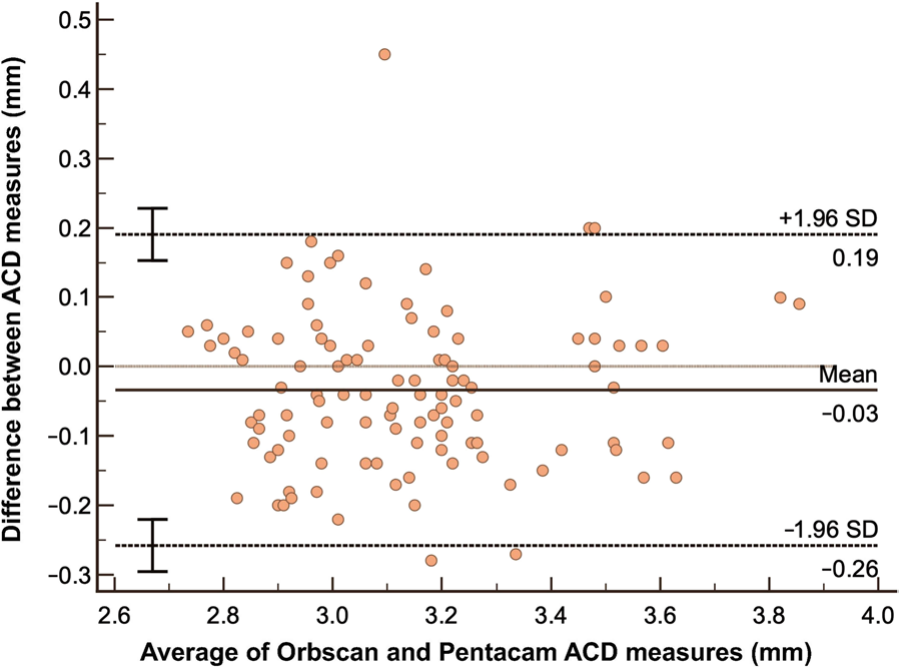
Bland-Altman analysis comparing ACD measurement using Orbscan versus Pentacam ACD Analysis. ACD, anterior chamber depth

In terms of ACD analysis, results showed that Orbscan versus IOLMaster has only a small systematic bias of just − 0.01 and has a very high correlation of 0.9288 which implies excellent agreement. The resulting LOA is from − 0.19 to 0.17 mm and almost all of the data points are within the LOA (Fig. 7). Orbscan versus Pentacam also has a small systematic bias of − 0.03, has an LOA of − 0.26 to 0.19 mm and a high correlation coefficient of 0.8906 which also implies good agreement (Fig. 8). Similarly, most of the data points are within the LOA. Between the two, IOLMaster has the closest systematic bias of 0.01, has a tighter LOA and higher correlation when compared with the Pentacam.

## Discussion

To determine accurate ICL sizing, both WTW and ACD measurements are needed. Various optical biometers and anterior segment devices can provide these measurements. However, most clinics will have only one and not a multiple array of devices at their disposal. The dilemma facing clinicians is whether their devices can provide raw data that result in an ideal vault post-implantation. If not, is there a mechanism or adjustment they can perform to increase the likelihood of their ICL surgeries achieving an ideal vault?

The Orbscan II, the longest standing device used for ICL sizing, is no longer in production. The IOLMaster 700 and Pentacam AXL are now more widely available and accessible to an increasing number of surgeons incorporating ICL implantation into their refractive practice. Therefore, it is important to evaluate the agreement in the recommended ICL sizing using the raw data obtained from these three machines.

Our study compared the raw data produced by these different anterior chamber devices. In addition, we obtained the WTW from a caliper to substitute for the WTW measurements provided by each machine to determine if this would improve the chances of achieving ideal vault measurement. A caliper is an inexpensive measuring tool that most clinics likely have at their disposal because it is used in other ophthalmic procedures. We used different combinations of raw data to calculate the recommended ICL size and compared which combinations were consistent and interchangeable. Lastly, we obtained the postoperative vault of ICL-implanted eyes and determined which combination had the highest percentage of eyes with the ideal vault.

Previous studies since 2004 have compared WTW corneal diameter measurements from different sets of devices (Table 5). The studies most similar to ours were by Baumeister et al. (100 eyes) evaluating the Orbscan, caliper, and IOLMaster 500 [18], Martin et al. (328 eyes) comparing Orbscan and IOLMaster 500, and Salouti et al. (101 eyes) comparing the Orbscan and Pentacam HR [19]. Our study is unique and timely because it compares four devices and the latest versions: the Orbscan II, Castroviejo caliper, newer swept-source IOLMaster 700, and the newer Pentacam AXL. Baumeister et al. found that measurement with the caliper and the Holladay-Godwin gauge is relatively imprecise and shows more significant variability and examiner dependence because the scale only estimates values between the marks. They likewise observed that the mean absolute WTW measurement value was 0.24 mm higher with the IOLMaster 700 than with the Orbscan II [18]. Martin et al. reported that the WTW distance measured by the IOLMaster 500 was 0.50 mm higher than the Orbscan II [20]. According to Salouti et al., the mean WTW distance reading with the Pentacam HR was 0.10 mm greater than that obtained with the Orbscan II. Although the difference showed statistical significance, it was clinically irrelevant. They concluded that the Orbscan II and Pentacam HR could be used interchangeably in clinical practice [19]. Lastly, in a study by Maged et al., they found that IOLMaster measurements were larger than the caliper measurements. Thus, a correction factor using a measurement reduction of –0.34 with the IOLMaster was suggested to attain the proper vaulting of the ICL. [29]

**Table 5:** Published literature on WTW corneal diameter of healthy eyes comparing different devices

Author	Year	Devices/study group	Subjects	Results
Baumeister et al. [18]	2004	Holladay-Godwin gauge, Zeiss IOLMaster, Orbscan	100 eyes (61 subjects)	The coefficient of inter-rater repeatability (COR) and LOA was 1.30 and − 0.82 to 1.77 mm for the caliper, 0.92 and − 0.82 to 1.01 mm for the Holliday Godwin gauge, 0.76 and − 0.75 to 0.79 mm for the Orbscan II and 0.50 and − 0.48 to 0.50 mm for the IOLMaster
Martin et al. [20]	2013	Group 1 comprised eyes with low myopia (< 6.00 D) Group 2 comprised moderately myopic eyes (6.00 to 12.00 D) Group 3 comprised extremely myopic eyes (> 12.00 D)	328 eyes (64 subjects)	Eyes with moderate (LOA − 1.04 to 0.02 mm; r = 0.69) and high myopia (LOA − 0.85 to − 0.19 mm; r = 0.94) had lower WTW diameters than eyes with low myopia (LOA − 1.02 to 0.05 mm; r = 0.76) measured with Orbscan and IOLMaster Orbscan topography provided less WTW distance than IOLMaster in myopic eyes, and thus the devices are not clinically interchangeable
Salouti et al. [24]	2009	Galilei, EyeSys, Orbscan II	74 eyes (37 subjects)	The best 95% LOA between devices were for the Galilei and the Orbscan II (− 0.72, 1.48; r = 0.40) The best 95% LOA between two eyes for each device were found with the Orbscan II (− 0.15, 0.17; r = 0.99) Results suggest that measurements made with the Orbscan II are smaller than those obtained with the EyeSys Corneal Analysis system and the Galilei
Salouti et al. [19]	2013	Pentacam HR, Orbscan II	101 eyes (101 subjects)	The mean difference between the Pentacam HR versus Orbscan IIz measurements was 0.10 ± 0.12 mm (95% confidence interval, 0.07–0.12, *P* < 0.001) The measurements were highly correlated (r = 0.948, *P* < 0.001) and the 95% LOAs for the Pentacam HR versus the Orbscan II were − 0.14 to 0.33 mm The observed differences in WTW distance readings between the Pentacam HR and the Orbscan IIz are clinically irrelevant, and the two devices can be used interchangeably in clinical practice
Guber et al. [25]	2015	Pentacam, Biograph Devices, HiScan Device	107 eyes (56 subjects)	The Allegro BioGraph measures of WTW were wider than those taken with the Pentacam (bias = 0.26 mm, *P* < 0.01) The repeatability STS measured with the HiScan was 0.39 mm, which was significantly reduced (0.15 mm) when the average of two measures was used Agreement between the horizontal WTW measures and horizontal STS estimates when bias was accounted for was r = 0.54 with the Pentacam and r = 0.64 with the BioGraph Large inter device bias was observed for WTW and STS measures
Fernández et al. [26]	2019	Orbscan and Keratograph	192 eyes of 192 subjects	Manual keratograph overestimated the WTW versus manual Orbscan in 0.13 ± 0.18 mm (*P* < 0.001) but not in the automated method comparison, 0.01 ± 0.19 mm (*P* = 0.58) Inter-examiner reproducibility was higher with manual Orbscan than with manual keratograph, and the intra-examiner *R* decreased with the average of two measures in both cases Probability of confounding sizing was higher with the increase of mean differences, the LOAs, and *R* WTW from 11.1 to 11.2 mm, 11.6 to 11.7 mm, and 12.3 to 12.4 mm resulted in higher PCS

*WTW* = white-to-white; *LOA* = limits of agreement; *STS* = sulcus to sulcus; *PCS* = probability of confusing sizing

Our study was consistent with findings in these previous studies that both IOLMaster and Pentacam had higher WTW measurements than Orbscan, which were statistically significant. The IOLMaster 700 had a higher WTW mean difference of 0.57 mm, while the Pentacam AXL was higher by 0.20 mm than the Orbscan. A unique observation was that the caliper had a mean WTW measurement 0.11 mm smaller than the Orbscan, which was also statistically significant. It should be noted that caliper measurements are quite subjective, observer-dependent, and may or may not be reproducible. What is important is that the examiner is mindful of being consistent with identifying landmarks in the limbus. We suggest that the same examiner performs these measurements in all patients to achieve consistency. In our practice, only one examiner performed all the caliper measurements. Our study results showed that measurements of WTW obtained from each device were significantly different from the Orbscan and each other, leading us to conclude that the four devices were not interchangeable with respect to WTW measurements.

The ACD is the second parameter crucial in calculating ICL size. There is no manual device that can measure ACD. The Orbscan, Pentacam and IOLMaster 700 are all capable of measuring the ACD starting from the endothelium to the anterior surface of the natural lens. A previous study comparing the ACD measurements of the Orbscan, IOLMaster 500, and Pentacam HR by Utine et al. showed that the ACD measurements of the IOLMaster 500 tended to be shorter by 0.06 mm while the Pentacam HR was larger by 0.05 mm compared to the Orbscan ACD measurements. The authors concluded that these differences did not result in noticeable differences in refractive outcomes, but no correlation analysis with the vault was performed [21]. In our study, pairwise comparison with the Orbscan ACD showed that both the IOLMaster 700 and Pentacam AXL had slightly larger measurements than the Orbscan, but only the Pentacam AXL mean difference of 0.03 mm demonstrated statistical significance.

We calculated the ICL size based purely on the WTW and ACD generated by each device. In addition, we substituted the caliper WTW, retained the ACD of each device, and generated another set of ICL size calculations. The objectives were to compare these six combinations and determine if they would produce similar ICL size recommendations to the arbitrary reference standard of purely using the Orbscan WTW and ACD.

Our study showed that four calculations generated the same ICL size as Orbscan WTW and ACD in almost 70% of eyes. These comparisons include pure Pentacam AXL WTW with ACD and caliper WTW in combination with either Orbscan, IOLMaster 700, and Pentacam AXL ACD raw data. Using purely IOLMaster 700 WTW and ACD will only yield the same size as Orbscan WTW and ACD in 29% of eyes. This is because the WTW from IOLMaster 700 was significantly larger. Using the caliper WTW instead of the IOLMaster WTW would improve the sizing to 64.5%, similar to the other combinations. This leads us to conclude that WTW is a more significant parameter that affects ICL sizing than ACD. Additionally, using the caliper WTW may be effective in improving the ICL sizing, especially for the IOLMaster 700 (Fig. 1).

Previous studies considered two postoperative vault ranges as reference or desired, either 0.25 mm to 0.75 mm [5–8] or 0.25 mm to 1.0 mm [9]. In our practice, we routinely measure postoperative vault with an anterior segment OCT. Using Orbscan WTW and ACD to calculate the ICL size, 70% of eyes in our study achieved a vault of 0.25 to 0.75 mm, while 91% had a vault between 0.25 to 1.00 mm.

Alsabaani et al. reported a 3.8% (22 of 787 eyes) incidence of ICL explantation. Twelve had high vaults and shallow anterior chambers, while 10 had low vaults resulting in excessive rotation or cataracts. There was no mention of the method of ICL size calculation and machine used to measure the vault [22]. In a study by Zeng et al., 16 out of the 616 myopic eyes had ICL exchange, with half having insufficient vault (≤ 0.1 mm) and the other half excessive vaulting (≥ 1.0 mm). They calculated the ICL size using measurements from Pentacam Scheimpflug pachymetry and a digital caliper under a surgical microscope. The vault was measured by 3D anterior segment optical coherence tomography (OCT) (3D OCT-2000, Topcon Corporation). [23]

In our study of 107 eyes, none had a vault of ≤ 0.1 mm, two had a vault < 0.25 mm, and seven had a high vault (≥ 1.0 mm). One eye had a vault measurement of 1.43 mm but with normal IOP. The patient underwent ICL exchange with a lower sized ICL (13.2 to 12.6 mm), which resulted in a post-exchange vault of 0.43 mm. Another patient had high vaults in both eyes (1.49 mm on the right and 1.53 mm on the left) and underwent bilateral ICL exchange with a smaller ICL size (13.2 to 12.6 mm). Two weeks after exchange surgery, the vault measured was 0.65 mm for both eyes. No complications or adverse events were encountered during or after ICL exchange procedures.

An important objective was to determine which device/devices would have the highest likelihood of matching the ICL size that achieved the desired vault range. With our point of reference using the Orbscan WTW and ACD, 91% achieved the desired vault range between 0.25 to 1.00 mm. The ICL size generated from the IOLMaster WTW and ACD had the lowest match rate (11%–15%) to our standard of care. The Pentacam WTW and ACD had the next lowest match percentage (41%–56%). However, when the caliper WTW measurement replaced the WTW measurement of both the IOLMaster 700 and Pentacam AXL, their match percentage increased to similar levels (60%–80%) as the Orbscan WTW/ACD and Caliper WTW/Orbscan ACD combinations. This supports our previous statement that using the caliper for measuring WTW may be effective in improving ICL sizing accuracy of the other newer devices (IOLMaster 700 and Pentacam AXL).

There are several limitations in our study. Firstly, interobserver differences in measuring WTW using the Castroviejo caliper may lead to inconsistencies. This can be remedied by assigning one examiner to do all the measurements because experience will increase skill and allow adjustments to be made more easily. Secondly, subjectivity in the designation of a range for the desired vault. Vaults outside this range do not necessarily mean this will lead to a complication or should be explanted or exchanged. A larger population size with longer follow-up period for measuring vault and monocular versus binocular involvement may lead to differences in the statistical significance of various measures. Lastly, the ultrasound biomicroscope is another device used to calculate ICL size, but this device produces a sulcus to sulcus measurement instead of a WTW measurement, so comparisons with the devices in our study are not appropriate.

## Conclusion

In conclusion, measurements of WTW obtained from the IOLMaster 700 and the Pentacam AXL were significantly different from the Orbscan II and each other. This led to differences in obtaining a consistent ICL size. The Orbscan WTW and ACD parameters have the highest percentage of eyes achieving vaults within the desired range. Purely using IOLMaster 700 and Pentacam AXL data would likely recommend a larger ICL size which may lead to an undesirable high vault. To compensate and improve sizing, combining the manual caliper WTW with either the IOLMaster 700 ACD or the Pentacam AXL ACD increases the success rate of achieving a desirable vault, thereby lessening the incidence of complications or explantations.

## Data Availability

Available from the corresponding author on reasonable request.

## Abbreviations

OCOS: Online calculation and ordering system
WTW: White-to-white
ACD: Anterior chamber depth
ICL: Implantable collamer lens
AL: Axial length
LOA: Limits of agreement
